# Supplementary material for: Sec16 alternative splicing dynamically controls COPII transport efficiency
Source: Nat Commun. 2016 Aug 5;7:12347. doi: 10.1038/ncomms12347 (PMC4980449; doi:10.1038/ncomms12347)
Supplement: Supplementary Information — Supplementary Figures 1-11 and Supplementary Table 1 [file ncomms12347-s1.pdf]

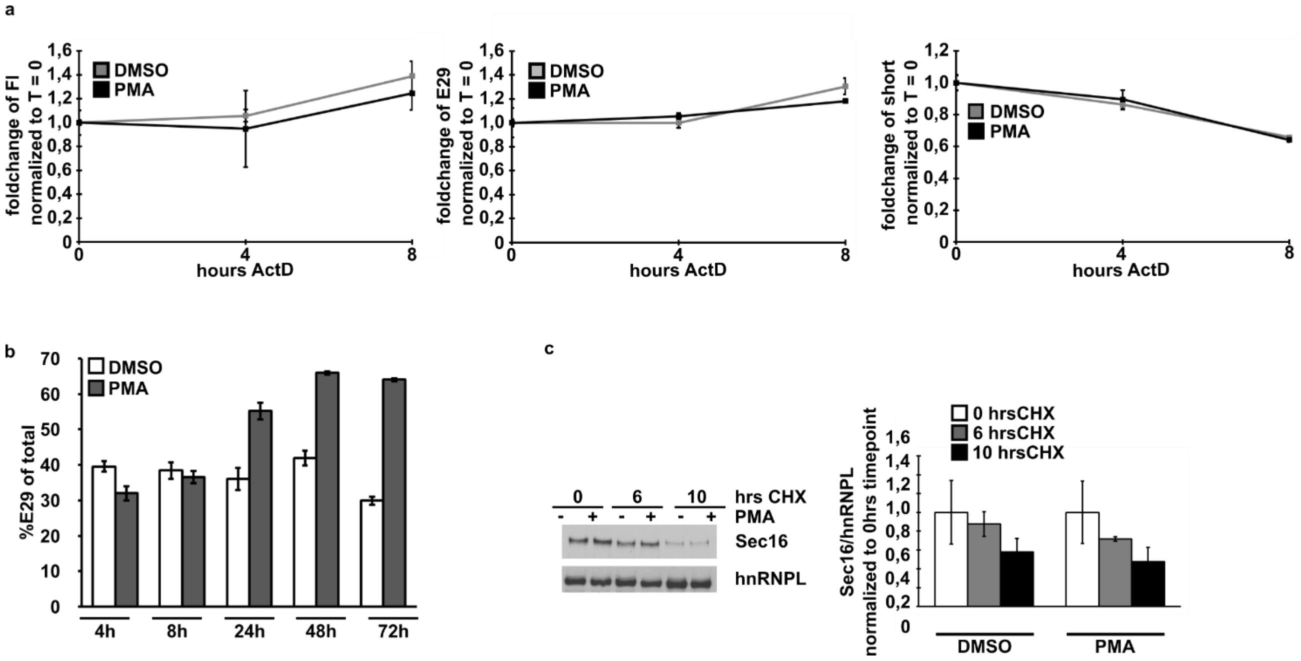

Supplementary Figure 1

(a) Sec16 mRNA stability is not regulated upon T cell activation. Resting and stimulated Jsl1 cells were treated with ActD and harvested for RNA extraction at the indicated timepoints. Radioactive splicing sensitive RT-PCR was performed as in Figure1. Phosphorimager analysis shows the average percentage of the Sec16 isoforms normalized to T = 0 +/- standard deviation of three independent experiments. Quantification of the E30 isoform is not shown as it is only barely detectable in stimulated cells. **(b) Sec16 E29 splicing time course shows an increase of the E29 isoform 24h post stimulation.** Phosphorimager analysis of radioactive RT-PCRs of RNA from resting and stimulated Jsl1 cells. Shown is the average percentage of E29 compared to total Sec16 RNA +/- standard deviation taken at the indicated timepoints (n = 3). **(c) Sec16 protein displays a half life of around 10h in resting and activated T cells.** To determine the half life of Sec16 protein, resting and stimulated Jsl1 cells were treated with CHX at a final concentration of 40 µg/ml, to block protein synthesis, and harvested at the indicated timepoints. Whole cell extracts were analyzed by Western blot using Sec16 antibody and hnRNPL as loading control. Quantification of Western blots show the average of three independent experiments +/- standard deviation. Values were normalized to loading control.

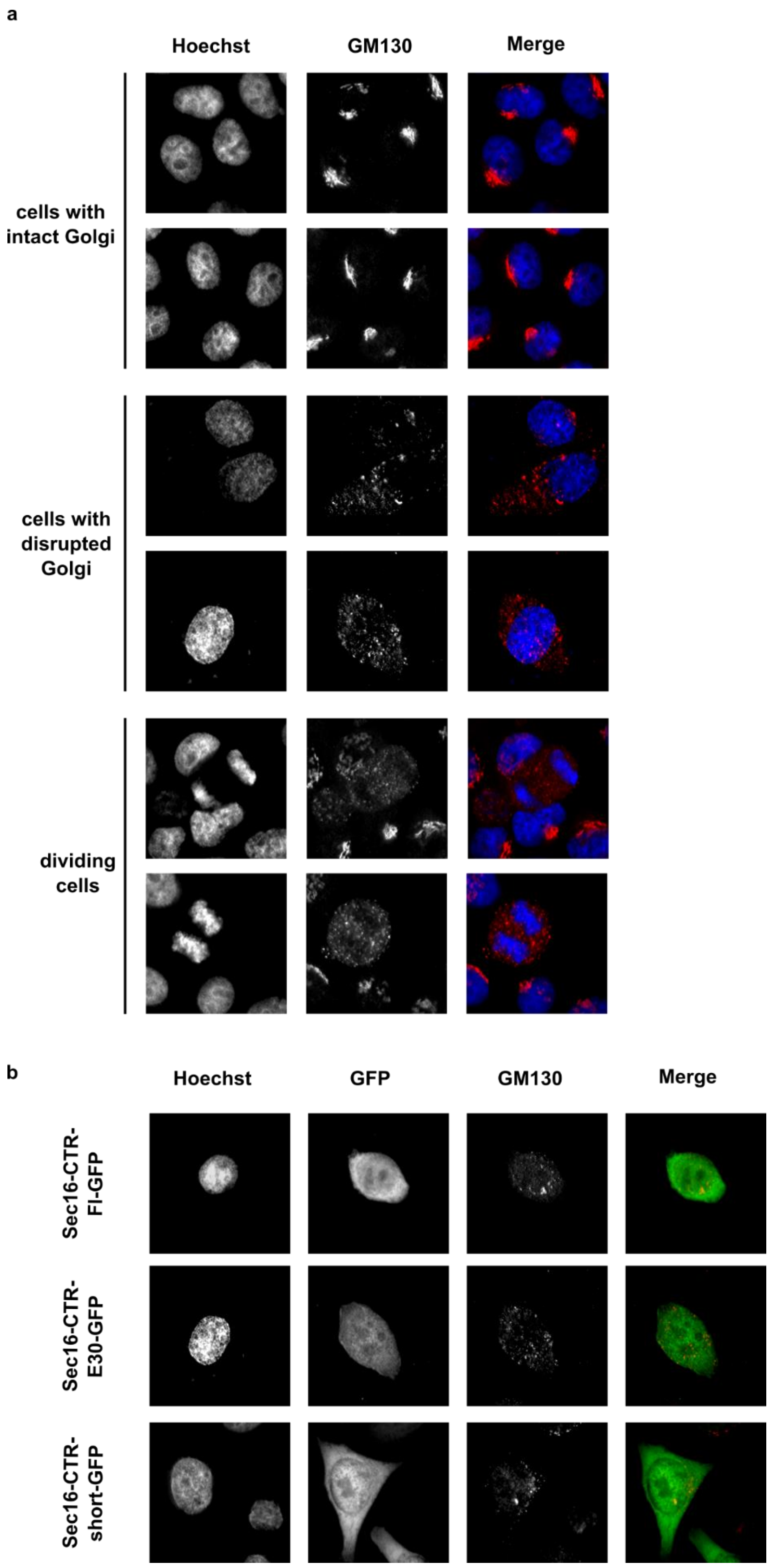

Supplementary Figure 2

(a) Definition of Golgi morphology. Top row shows Golgi staining in untransfected HeLa cells, defining intact Golgi morphology. Middle row shows a dispersed Golgi. Lower row shows Golgi staining in dividing cells. **(b) Overexpression of Sec16-CTRs in HeLa cells.** Fluorescence pictures show cytoplasmic localization of GFP tagged Sec16 CTRs.

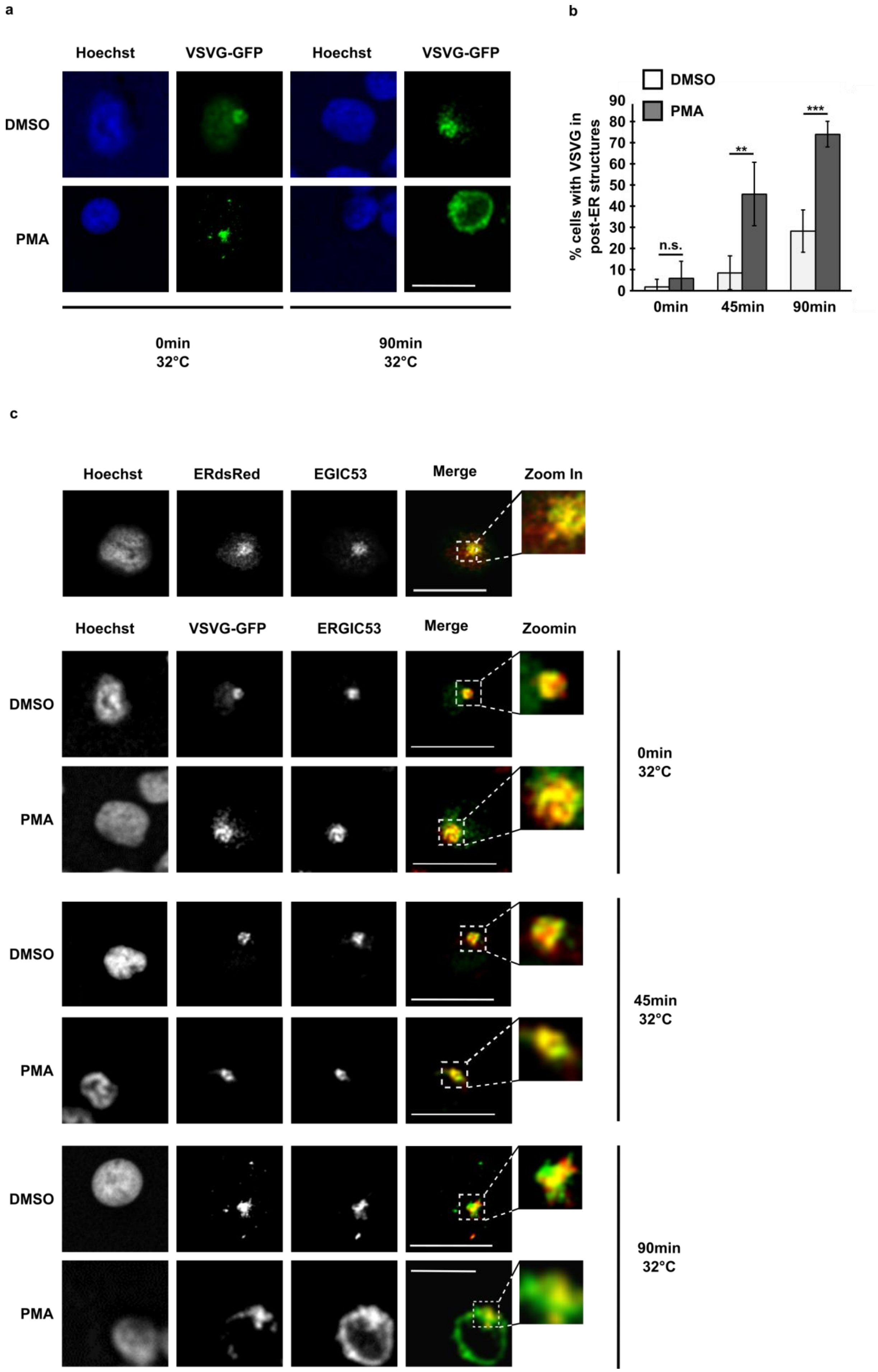
a
Hoechst
VSVG-GFP
Hoechst
VSVG-GFP
DMSO
PMA
0min
32°C
90min
32°C
b
DMSO
PMA
% cells with VSVG in post-ER structures
90
80
70
60
50
40
30
20
10
0
n.s.
**
***
0min
45min
90min
c
Hoechst
ERdsRed
EGIC53
Merge
Zoom In
Hoechst
VSVG-GFP
ERGIC53
Merge
Zoomin
DMSO
PMA
0min
32°C
DMSO
PMA
45min
32°C
DMSO
PMA
90min
32°C

Supplementary Figure 3

(a) Activated T cells show increased ER export efficiency. Representative Immunofluorescence pictures of Jsl1 cells stably expressing the ts045-VSVG-GFG variant either directly after heatshock or 90min after shifting cells to 32°C. The Export assay was performed as described in Material and Methods. Scale bar represents 20µm.

(b) Cells described in (a) were counted regarding the location of VSVG-GFP. Two individual VSVG-GFP expressing cell lines were analyzed in two independent experiments with an average of 10 cells per time point and condition.

(c) VSVG clusters in ERGIC53-positive structures upon heatshock. Top panel: Jsl1 cells were transfected with a plasmid encoding dsRed fused to an ER retention signal. Cells were shifted to 40°C overnight and fixed for immunofluorescence the following day. Cells were stained with the ERGIC marker ERGIC53. All others: Stable VSVG-GFP expressing Jsl1 cells were treated with PMA or DMSO as control and exposed to 40°C overnight and analyzed for reporter protein localization via fluorescence microscopy. Shown is a costaining with the ERGIC-marker ERGIC53, which colocalizes with the ER under these conditions (top panel). Scale bar represents 20µm.

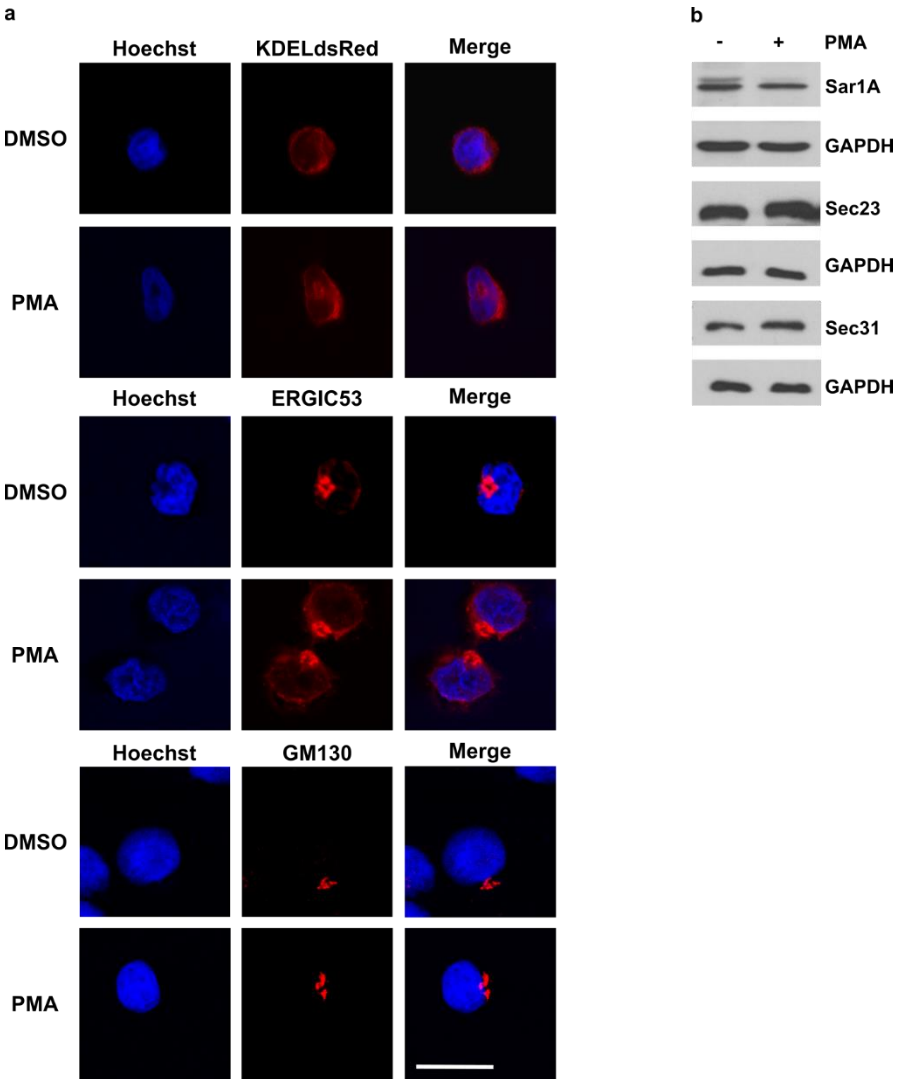

Supplementary Figure 4

(a) The ER, ERGIC and Golgi do not change their morphology upon T cell activation. Fluorescence pictures of resting and stimulated Jsl1 cells analyzing compartments of the early secretory pathway as indicated. Scale bar represents 20 µm. **(b) Western blot analysis of resting and stimulated Jsl1 cells.** Marginal changes of the total protein level of Sar1A, Sec23A and Sec31 compared to GAPDH loading control were observed. We repeatedly observe a higher migrating Sar1 band in unstimulated Jsl1 T cells. This could represent a posttranscriptional or posttranslational modification on Sar1A, or Sar1B, as the antibody also recognizes Sar1B (unpublished observation). For the purpose of this study, this issue was not further investigated.

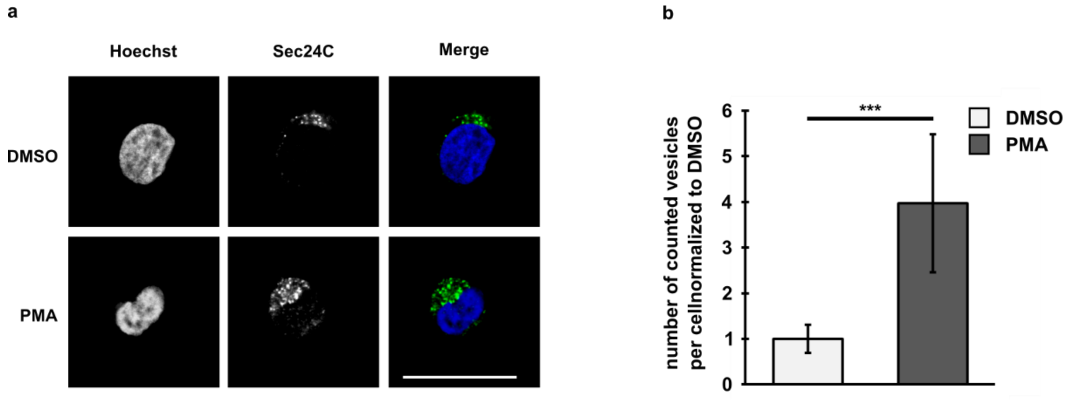

Supplementary Figure 5

(a) Immunofluorescent staining of Sec24C. Representative fluorescent pictures of resting and activated Jsl1 cells. Scale represents 20 µm. (b) **Number of COPII vesicles increases upon stimulation.** Quantification of Sec24C stained COPII vesicles in cells described in (a). For DMSO 18 cells were analyzed, for PMA 24 cells. Raw numbers of vesicles are for DMSO 13,6 +/- 4,26; for PMA 54 +/- 20,58. P = 7.9×10^{-10}.

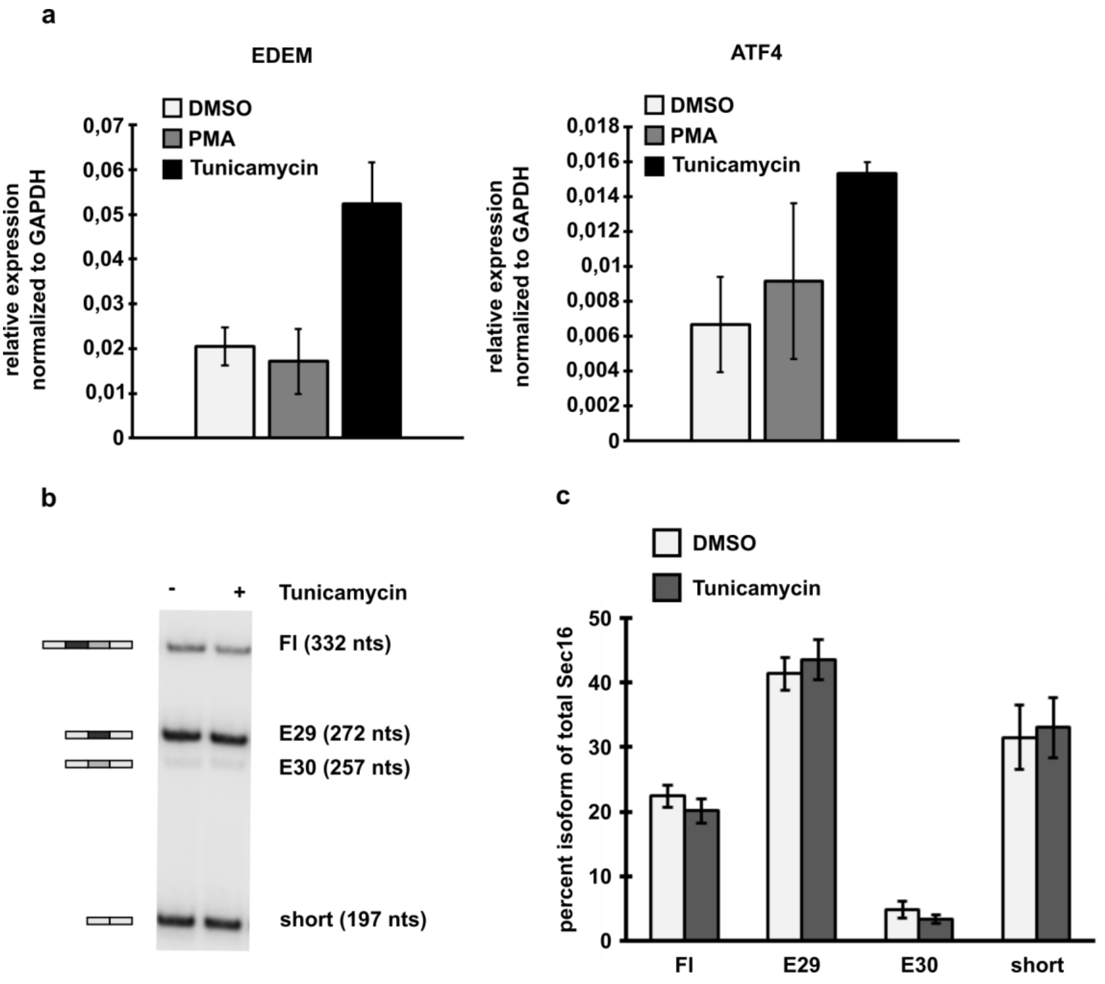

Supplementary Figure 6

(a) PMA treatment does not induce ER stress. Jsl1 cells were treated 48 hrs with either PMA or DMSO as solvent control. As positive control cells were treated with 1µg/ml Tunicamycin overnight. RNA was extracted and expression of two ER-stress markers was analyzed by RT-qPCR (EDEM and ATF4).Samples from 3 independent experiments were measured in triplicates. **(b) Sec16 splicing is not altered under ER stress.** Jsl1 cells were treated with 1µg/ml Tunicamycin overnight to induce ER stress. RNA was extracted and analyzed in a radioactive splice sensitive RT-PCR (n = 6).

a

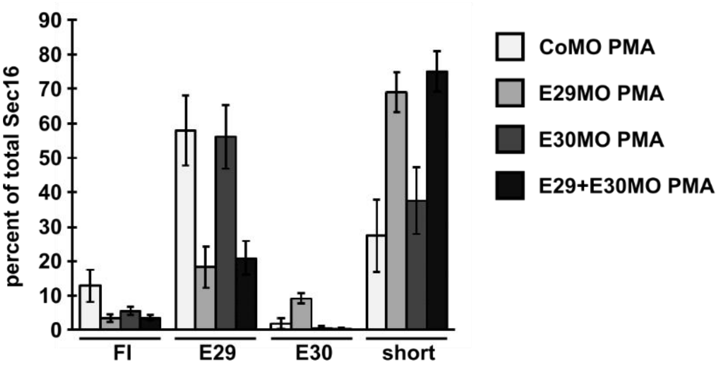

b

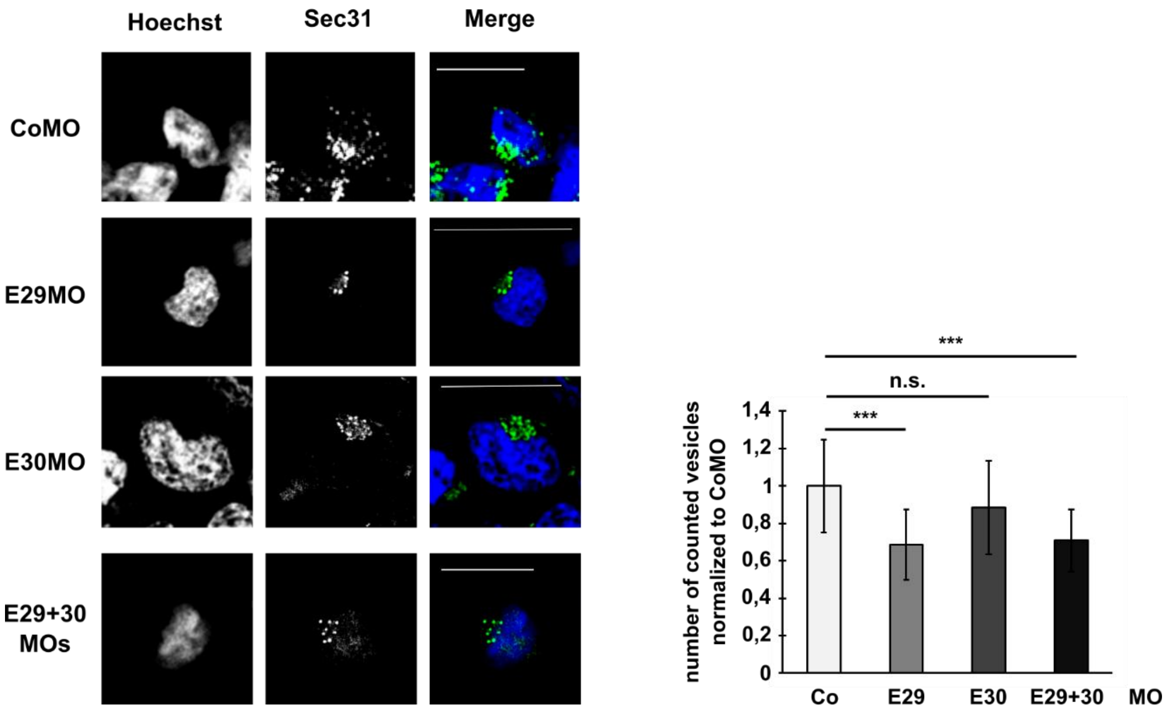

Supplementary Figure 7

(a) Influence of MO transfection on Sec16 splice pattern in stimulated T cells. Stimulated Jsl1 cells from independent MO transfection were analyzed for Sec16 splicing via radioactive RT-PCR. Shown is the mean percentage of the isoforms +/- standard deviation. **(b) Induced E30 skipping has no significant influence on ERES number.** Representative immunofluorescence pictures of stimulated MO transfected Jsl1 cells. At least 25 cells per condition were analyzed for ERES by Sec31 staining. Shown is the average number of ERES normalized to CoMO +/- standard deviation.

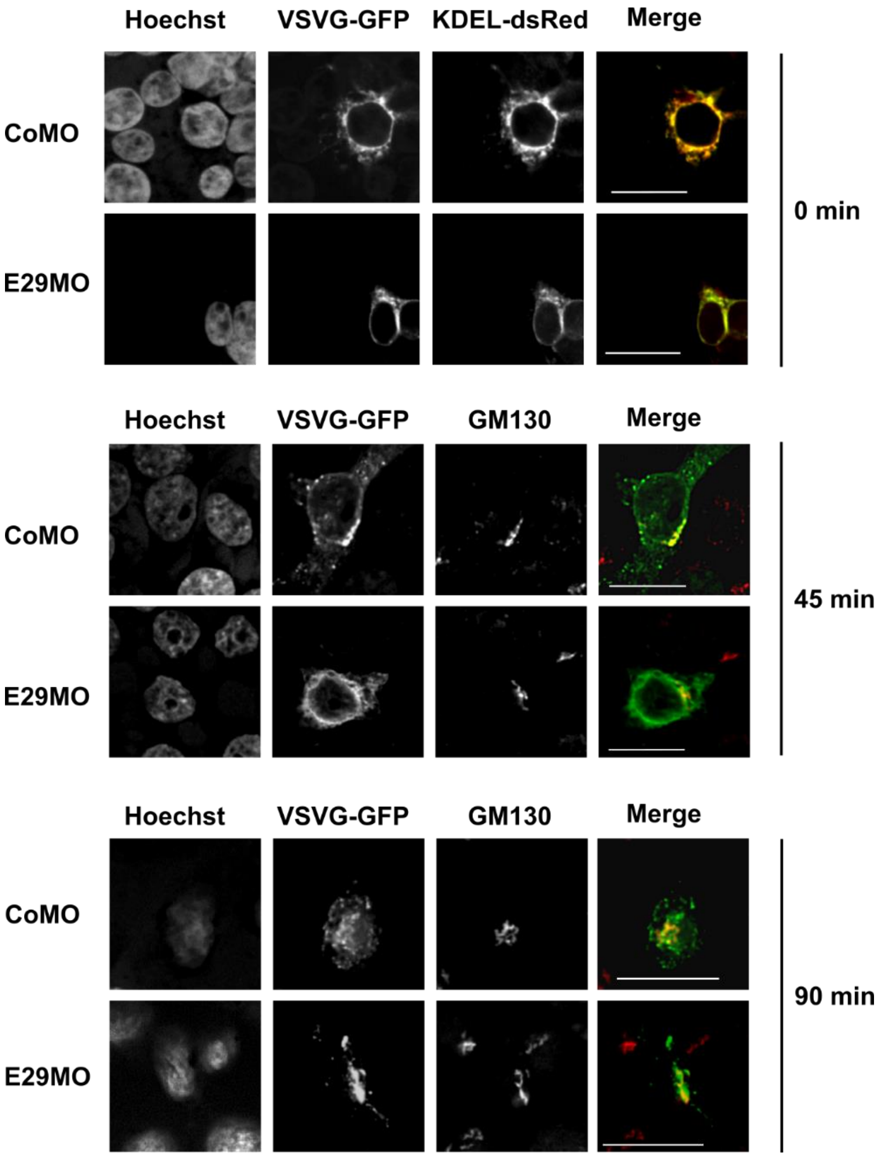

Supplementary Figure 8

The Sec16 E29MO reduces ER export efficiency. In support to Figure 5e pictures of every timepoint are shown with costainings of organell markers. For 0 min ER is costained (in red) for 45 and 90 min the Golgi is costained (in red). Scale bar represents 20µm.

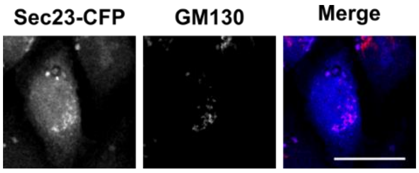

Supplementary Figure 9

Expression of Sec23-CFP alone has no influence on Golgi morphology. Representative fluorescence pictures of HeLa cells expressing Sec23-CFP display intact Golgi morphology as marked by GM130 staining. Scale bar represents 20µm.

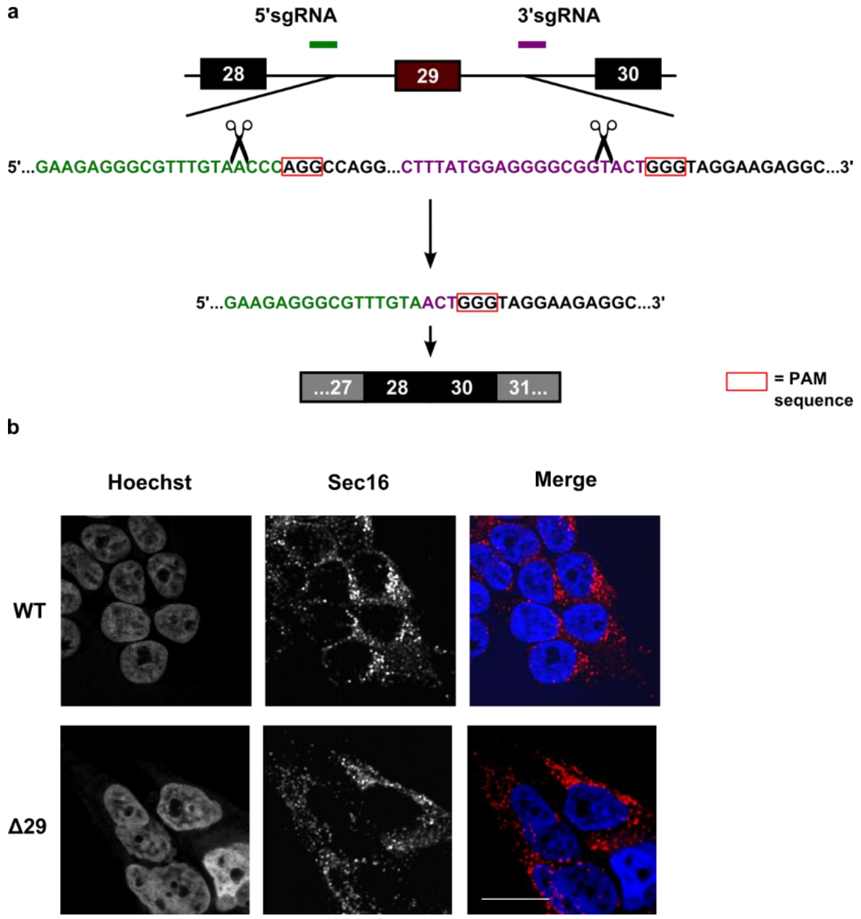

Supplementary Figure 10

(a) Scheme illustrating the CRISPR/Cas9 approach. Cas9 nuclease was guided to intronic sequences up- and downstream of Sec16 E29. Genomic sequence of the resulting deletion was obtained by sequencing of PCR products. **(b) No significant difference in Sec16 staining in WT and ΔE29 cells.** WT and ΔE29 Hek293 cells were fixed and stained for endogenous Sec16. Scale bar represents 20µm.

Supplementary Figure 11

Original scans of Western Blots shown in the manuscript. Red rectangle marks the part that is shown. In some cases the membrane was cut and therefore does not contain the full size range.

Fig. 1d 6% SDS-PAGE (top: αSec16, bottom: αhnRNPL)

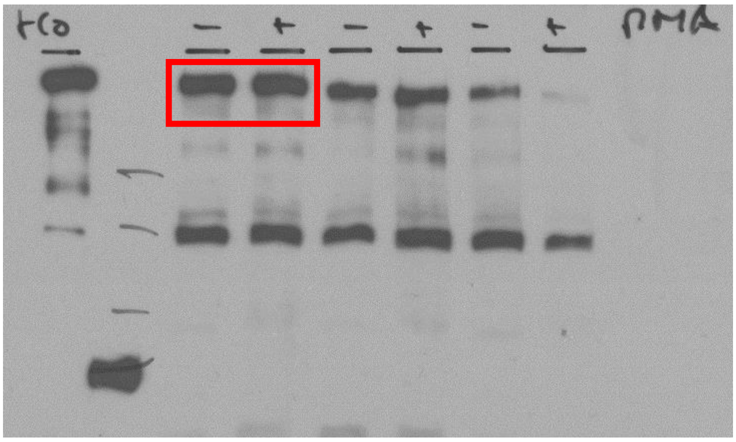

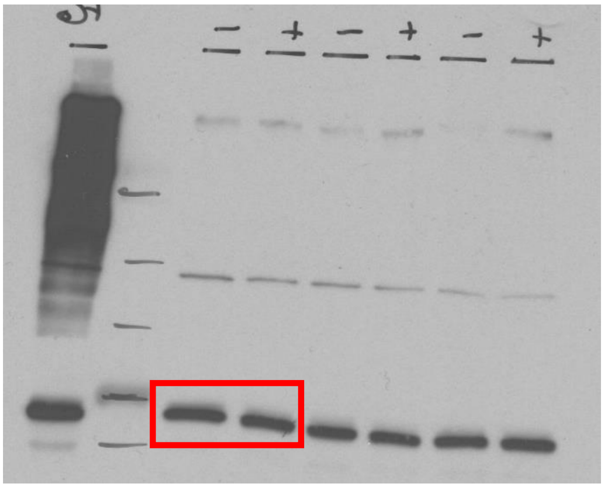

Fig. 1d 4-12% NU-PAGE (top: αSec16, bottom: αhnRNPL)

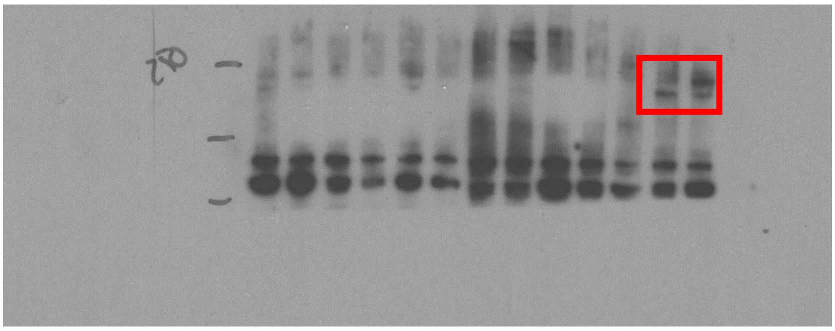

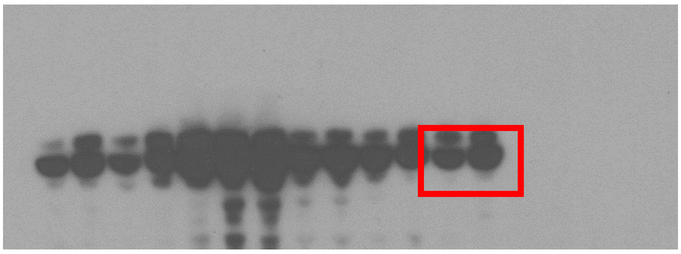

Fig. 2c (left αGFP, right αGAPDH, after αGFP)

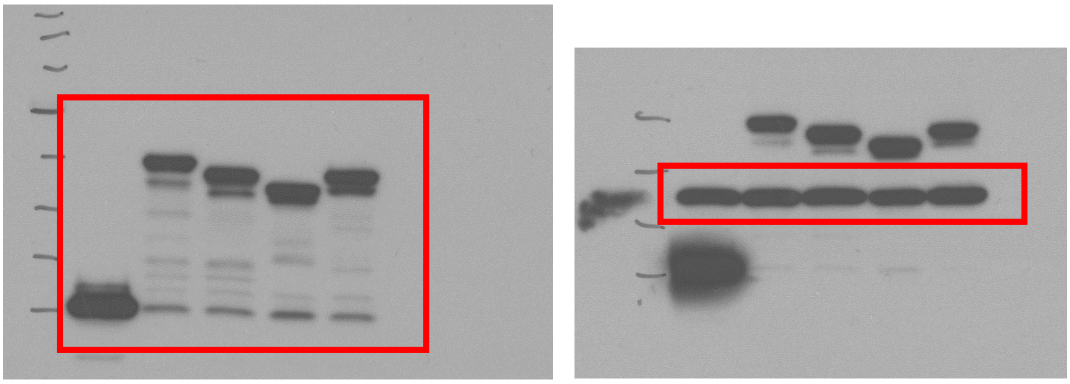

Fig. 4d 6% SDS-PAGE (left αSec16, right αhnRNPL)

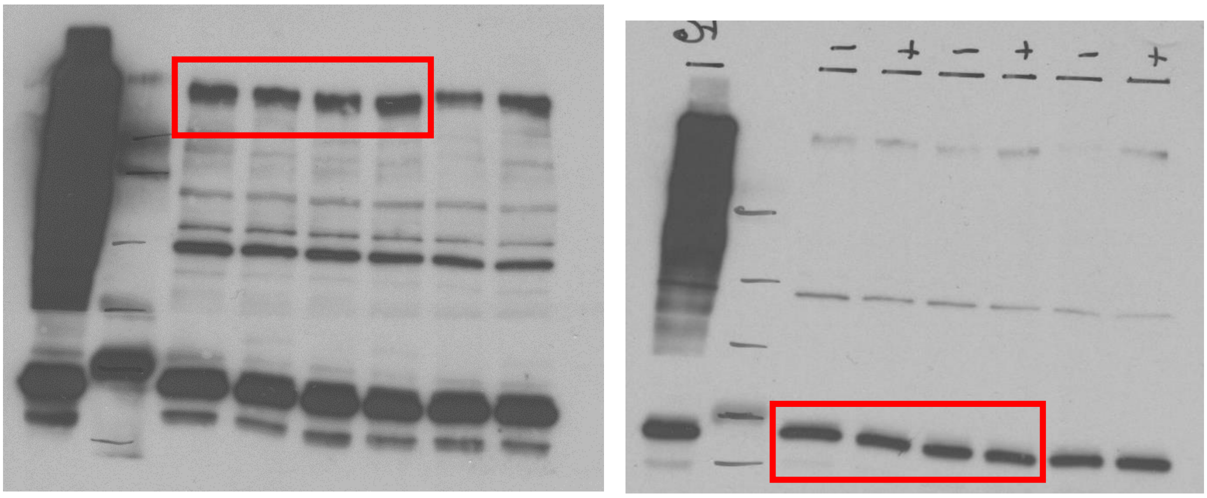

Fig. 4d 4-12% NU-PAGE (top αSec16, bottom αhnRNPL)

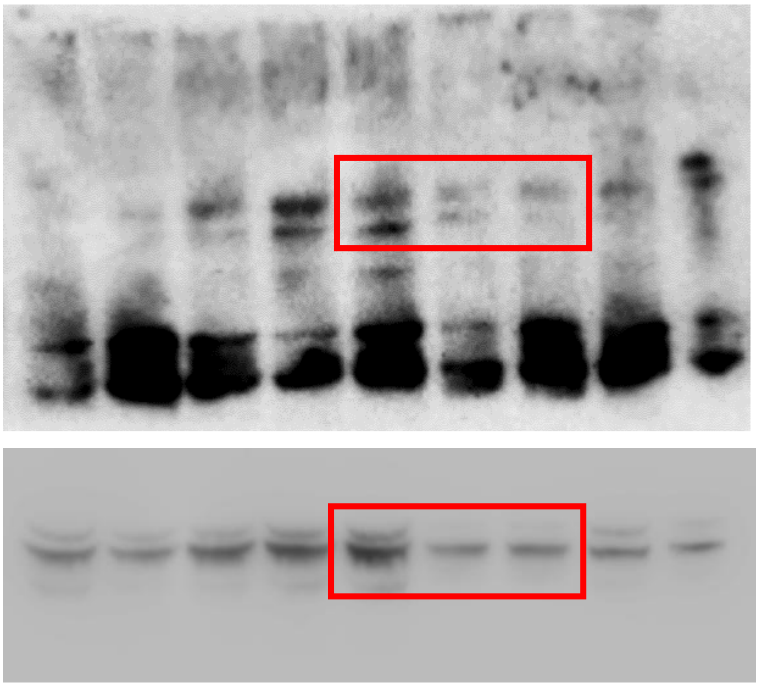

Fig. 6a

Sar1A - Input (left αGFP, right αFLAG)

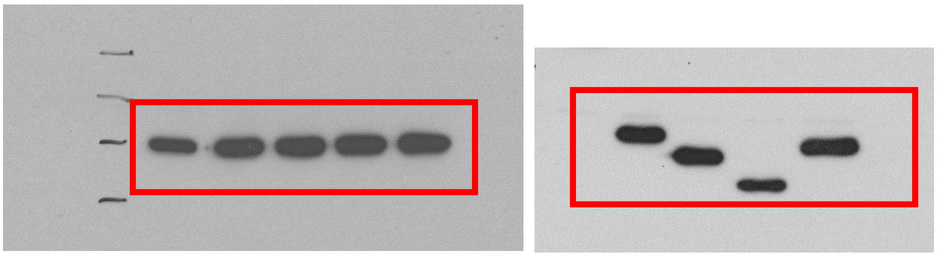

Sar1A - IP (left αGFP, right αFLAG)

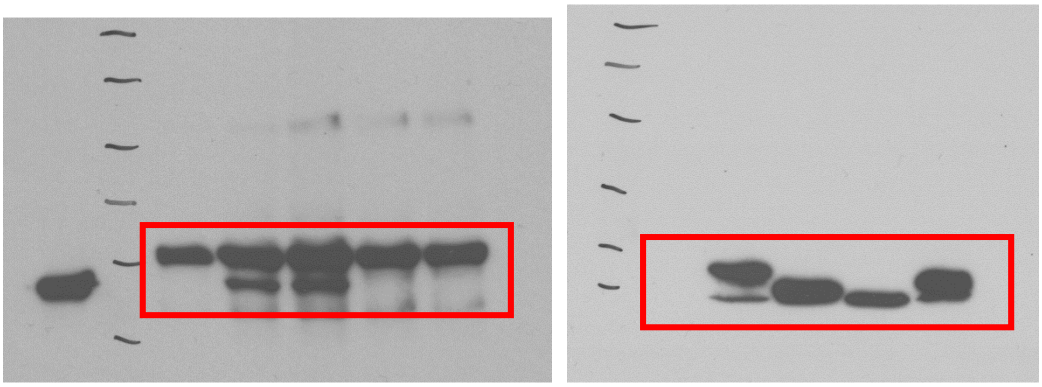

Sar1B – Input (top αGFP, bottom αFLAG)

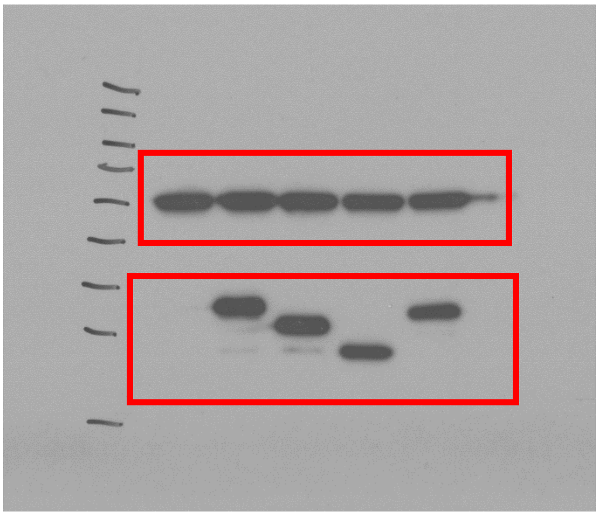

Sar1B – IP (left αGFP, right αFLAG)

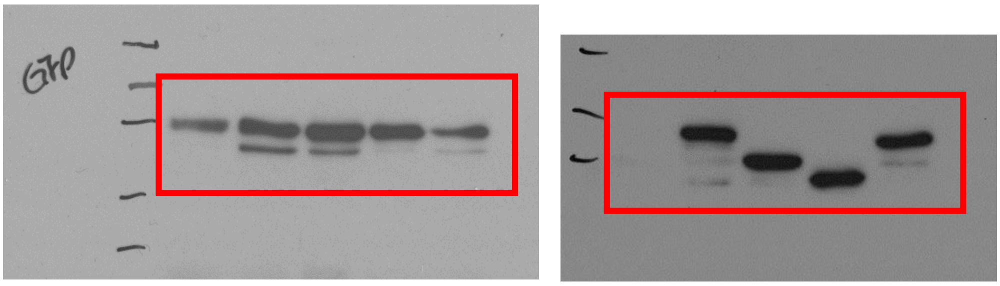

Sec12 – Input (top αGFP, bottom αFLAG)

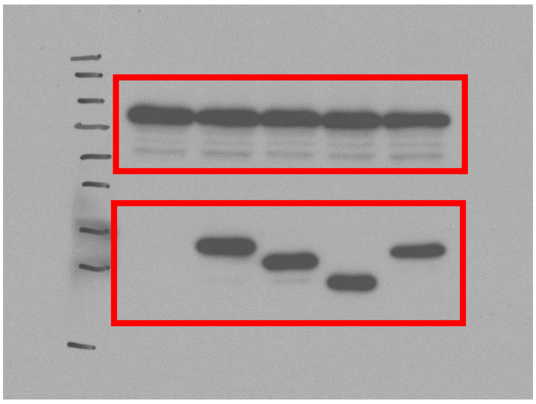

Sec12 – IP (top αGFP, bottom αFLAG)

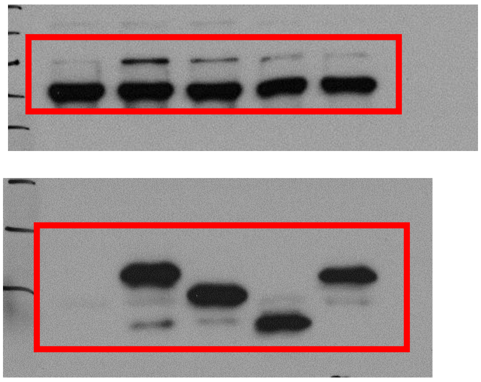

Sec23 – Input (top αGFP, bottom αFLAG)

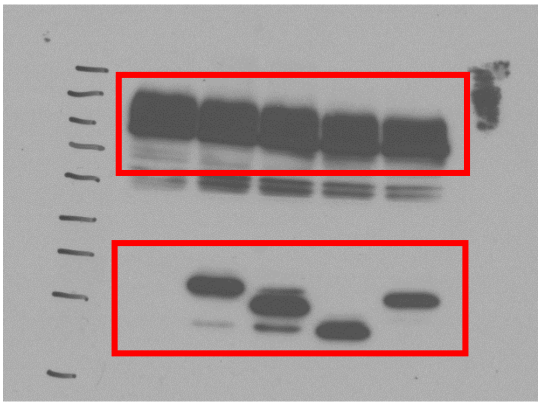

Sec23 – IP (top αGFP, bottom αFLAG)

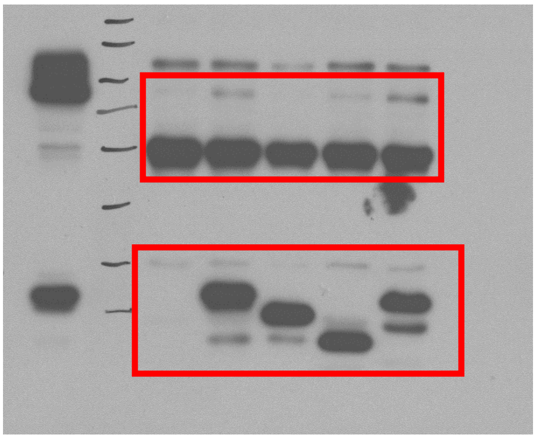

Fig. 8b (top αSec16, bottom αhnRNPL)

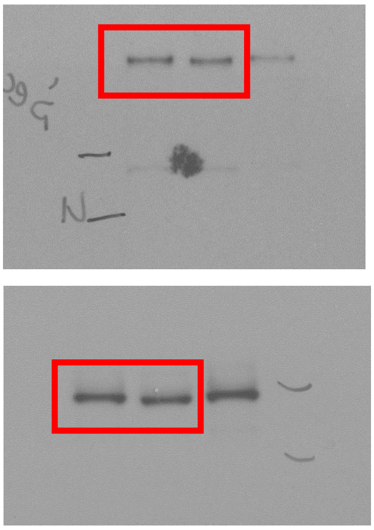

Fig. 8e

0 min (top αGFP, bottom αhnRNPL)

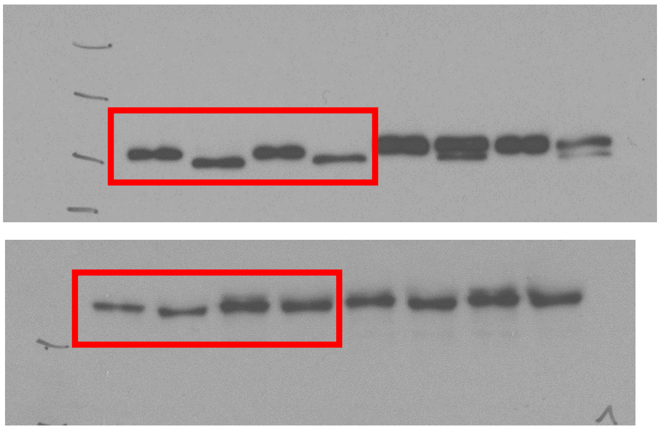

45 min (top αGFP, bottom αhnRNPL)

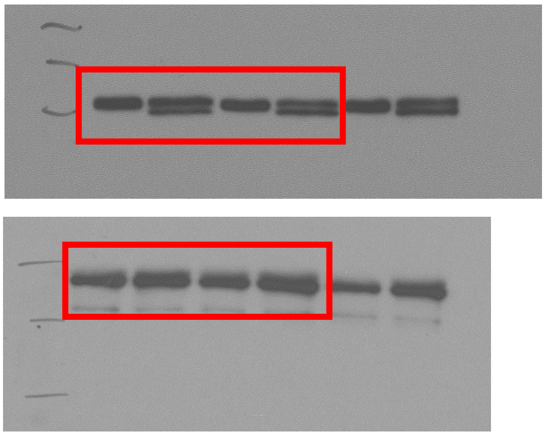

90 min (top αGFP, bottom αhnRNPL)

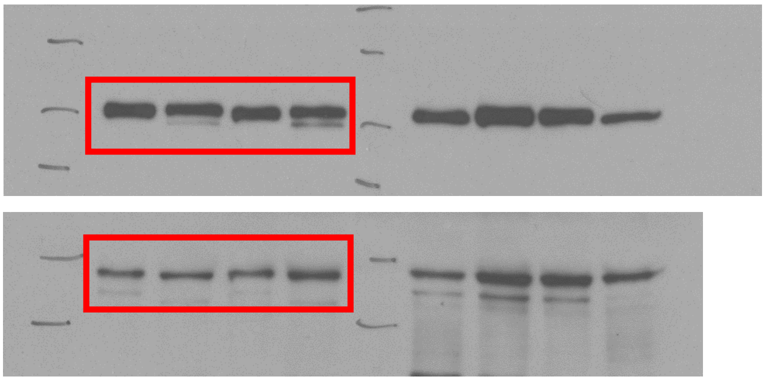

Supplementary Figure 1c (left αSec16, right αhnRNPL)

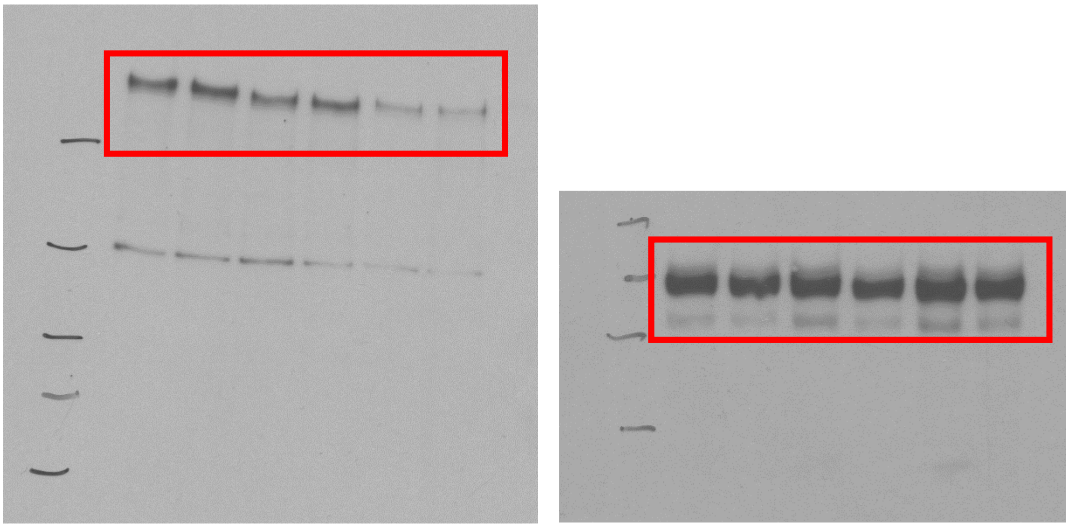

Supplementary table 1

List of oligonukleotides used in this study for cloning and qPCR. A description and the sequence of the respective primers is given below.

Cloning primer		
Insert	description	Sequence
Sec16 CTRs		
Fl and short	Sec16-CTR-XhoI-fw	GCCTCGAGACCATGGAGAAGA AAGCCCCGCCCC
Fl and short	Sec16-CTR-BamHI-rev	GCGGATCCGTTCAGCACCAGGTGCTTCCTC
Sec16-Fl and short CTRs were PCR amplified using the same primers and identified by their bp size.		
E30	Sec16-E30-fw	CTTTCGCGCTGTAGTTCAATG
E30	Sec16-E30-rev	GCTCCTACAGCGCGAAAGCTTGGGCTCTGGGGCAG
E29	sec16-E29-fw	GCTCCTGGCGACCTCCCTGC
E29	Sec16-E29-rev	GGTCGCCAGGAGCCTCTCCTCCCTGGG
Sec16-E29 and E30 CTRs were cloned via two-step-PCR		
cytoplasmic Sec12	Sec12-XhoI-fw	GCCTCGAGATGGGCCGGCGCCGGGCGC
cytoplasmic Sec12	Sec12-BamHI-rev	CGGGATCCCCGTGAGGGCAACAGATGCAGC
Sec23	Sec23-XhoI-fw	AGCTCGAGATGACAACCTATTTGGAATTCATTCAAC
Sec23	Sec23-BamHI-rev	GCGGATCCAGCAGCACTGGACACAGCAAGTTTCTTC
Sar1A	Sar1A-NdeI-fw	CGCATATGTCTT TCATCTTTGAGTGGATCTAC AATGG
Sar1A	Sar1A-BamHI-rev	GCGGATCCGTCAATATACTGGGAGAGCCAGCGG
Sar1B	Sar1B-NdeI-fw	CGCATATGTCCT TCATATTTGATTGGATTTAC AGTGG
Sar1B	Sar1B-BamHI-rev	GCGGATCCATCAATGTACTGTGCCATCCAGCGGAAGC
Sar1 A and B were first cloned into pGADT7, cut and ligated into pCMV-N3-GFP.		
PCR primer detecting CRISPR/Cas9 mediated Sec16 E29 deletion		
	Sec16-DE29-fw	GCACACACAGGCACCTGGTAG
	Sec16-DE29-rev	GAACACCACCTTCGAGGTGCTC

qPCR primer		
hGAPDH-fw	house keeping gene	CTTCGCTCTCTGCTCCTCCTGTTCG
hGAPDH-rev	house keeping gene	ACCAGGCGCCCAATACGACCAAAT
hATF4-fw	ER stress marker	GTTCTCCAGCGACAAGGCTA
hATF4-rev	ER stress marker	ATCCTGCTTGCTGTTGTTGG
hEDEM-fw	ER stress marker	CAAGTGTGGGTACGCCACG
hEDEM-rev	ER stress marker	AAAGAAGCTCTCCATCCGGTC
